# Diversity of the association of serum levels and genetic variants of MHC class I polypeptide-related chain A with liver fibrosis in chronic hepatitis C

**DOI:** 10.18632/oncotarget.15941

**Published:** 2017-03-06

**Authors:** Chung-Feng Huang, Ching-I Huang, Ming-Lun Yeh, Shu-Chi Wang, Kuan-Yu Chen, Yu-Min Ko, Ching-Chih Lin, Yi-Shan Tsai, Pei-Chien Tsai, Zu-Yau Lin, Shinn-Cherng Chen, Chia-Yen Dai, Jee-Fu Huang, Wan-Long Chuang, Ming-Lung Yu

**Affiliations:** ^1^ Hepatobiliary Division, Department of Internal Medicine, Kaohsiung Medical University Hospital, Kaohsiung Medical University, Kaohsiung, Taiwan; ^2^ Faculty of Internal Medicine, School of Medicine, College of Medicine, Kaohsiung Medical University, Kaohsiung, Taiwan; ^3^ Department of Occupational Medicine, Kaohsiung Medical University Hospital, Kaohsiung Medical University, Kaohsiung, Taiwan; ^4^ Department of Preventive Medicine, Kaohsiung Medical University Hospital, Kaohsiung Medical University, Kaohsiung, Taiwan; ^5^ Institute of Biomedical Sciences, National Sun Yat-Sen University, Kaohsiung, Taiwan; ^6^ Liver Center, Division of Gastroenterology, Massachusetts General Hospital, Harvard Medical School, Boston, MA, USA

**Keywords:** MICA, SNP, sMICA, liver fibrosis, CHC

## Abstract

**Background/Aims:**

Genetic variants of MHC class I polypeptide-related chain A (MICA) at rs2596542 have been associated with hepatocellular carcinoma. The linkage between serum MICA (sMICA) and liver fibrosis in chronic hepatitis C is elusive.

**Results:**

Linear regression analysis revealed that sMICA were independently correlated to α-fetoprotein (β: 0.149; 95% confidence interval [CI]: 0.001, 0.003; *P* = 0.007)and MICA rs2596542 GG genotype (β: 0.209; 95% CI: 0.153, 0.483; *P* < 0.001). While patients were stratified by MICA genetic variants, advanced fibrosis was the only factor independently correlated to sMICA among A allele carriers (β: 0.234; 95% CI: 0.107, 0.543; *P* = 0.004) but not among non-A allele carriers. Logistic regression analysis revealed that factors associated with advanced liver fibrosis was sMICA (OR/CI: 2.996/1.428–6.287, *P* = 0.004) and platelet counts (OR/CI: 0.988/0.982–0.994, *P* < 0.001) in MICA rs2596542 A allele carriers. sMICA > 50 pg/mL provided a positive predictive value of 72 % in predicting advanced liver fibrosis (F3-4) and of 90% in significant fibrosis (> F2) in MICA rs2596542 A allele carriers.

**Materials and Methods:**

Serum level and single nucleotide polymorphism at rs2596542 of MICA were tested for the association with liver fibrosis in 319 biopsy proven chronic hepatitis C patients.

**Conclusions:**

Levels of sMICA were highly correlated to liver disease severity in chronic hepatitis C patients who carried the MICA rs738409 A allele. Patients possessing the genetic predisposition had a higher likelihood of progressed liver fibrosis if they expressed higher sMICA levels.

## INTRODUCTION

Chronic hepatitis C (CHC) infection is one of the leading causes of end sage liver diseases including hepatocellular carcinoma (HCC) [1]. Fibrogenesis is a critical pathophysiological element for clinical deterioration in CHC patients. The rate of liver fibrosis progression varies and the major determinants include viral factors (ex. hepatitis B virus [HBV] and human immunodeficiency virus [HIV] co-infection), environmental factors (ex. alcohol) and host factors (ex. diabetic mellitus, age, age of infection, steatosis and host genetics [2–5]).

When it comes to host genetics, a genome-wide association study (GWAS) has confirmed that the single nucleotide polymorphism (SNP) rs2596542 of MHC class I polypeptide-related chain A (MICA) was significantly associated with hepatitis C virus (HCV) related HCC [6]. MICA is the ligand for natural killer group 2D (NKG2D). It activates natural killer cells and CD8+ T cells, which in turn express anti-tumor effects. However, high expression soluble MICA (sMICA) in the circulation might down-regulate NKG2D expression. It may further disturb NKG2D-mediated antitumor immunity. We have demonstrated the impact of the SNP and its serum levels on HCC occurrence upon a HCV post-treatment cohort [7]. In view of liver disease severity, it has been suggested that HCV-related liver cirrhosis has higher sMICA levels compared to uninfected subjects [8]. However, whether the sMICA could serve as a biomarker of liver fibrosis among CHC patients has never been explored. Furthermore, the expression of sMICA varies between subjects who carry different MICA rs2596542 genotypes [6, 8]. It is imperative to explore the issue by judging patients with different genetic backgrounds. In the current study, we aimed to elucidate the association of sMICA with HCV-related liver fibrosis in patients with different genetic predispositions.

## RESULTS

### Patient characteristics

The basic demographical, virological, and clinical features of the 319 patients are shown in Table 1. The mean patient age was 52.9 ± 11.3 years. Males accounted for the 51.7% of the population. The mean HCV RNA levels were 5.55 ± 0.96 log IU/ml and 65.8% of the patients were infected with HCV genotype 1. Patients with advanced fibrosis (F3-4) accounted for 49.2% of the population. The mean sMICA level was 1.50 ± 0.76 log pg/mL, and the proportion of patients with MICA rs2596542 A allele carriage was 49.2 % (*n* = 157). The MICA rs2596542 SNP distribution and sMICA level did not differ between patients with or with advanced liver disease among the entire population.

**Table 1:** Basic characteristics of the patients

	All patients (*n* = 319)	F0-2 (*n* = 162)	F3-4 (*n* = 157)	*P* value
Age (years, mean ± SD)	52.9 ± 11.3	51.0 ± 11.9	54.8 ± 10.2	0.003
Male gender, *n* (%)	165 (51.7)	85 (52.5)	80 (51.0)	0.79
Body weight (kg, mean ± SD)	66.8 ± 11.8	65.8 ± 11.5	67.9 ± 12.1	0.11
DM, *n* (%)	56 (17.6)	23 (14.2)	33 (21.0)	0.11
Platelet count (× 10^3^ *u*/L, mean ± SD)	160 ± 64	184 ± 62	136 ± 57	< 0.001
*r*-GT (U/L,mean ± SD)	68.8 ± 57.8	58 ± 52	80 ± 61	0.001
AST (IU/L, mean ± SD)	104 ± 59	96 ± 54	112 ± 64	0.02
ALT (IU/L, mean ± SD)	150 ± 97	151 ± 96	149 ± 97	0.84
α-fetoprotein (ng/mL, mean ± SD)	21.2 ± 59.8	11.2 ± 15.0	31.4 ± 82.7	0.003
HCV genotype 1, *n* (%)	210 (65.8)	111 (68.5)	99 (63.1)	0.30
HCV RNA (log IU/mL, mean ± SD)	5.55 ± 0.96	5.63 ± 0.99	5.46 ± 0.93	0.11
MICA rs2596542 A allele, *n* (%)	157 (49.2)	73 (45.1)	84 (53.5)	0.13
sMICA (log pg/mL,mean ± SD)	1.50 ± 0.76	1.44 ± 0.76	1.57 ± 0.76	0.10

Note: HCV, hepatitis C virus;MICA: MHC class Ipolypeptide-related chain A;sMICA: serum MICA level; *r*-GT: *r*-glutamyl transferase; SD: standard deviation; DM: diabetes mellitus; AST: aspartate aminotransferase; ALT: alanine aminotransferase.

### Association of sMICA with liver fibrosis in patients with different MICA rs2596542 genotypes

**Figure 1:**
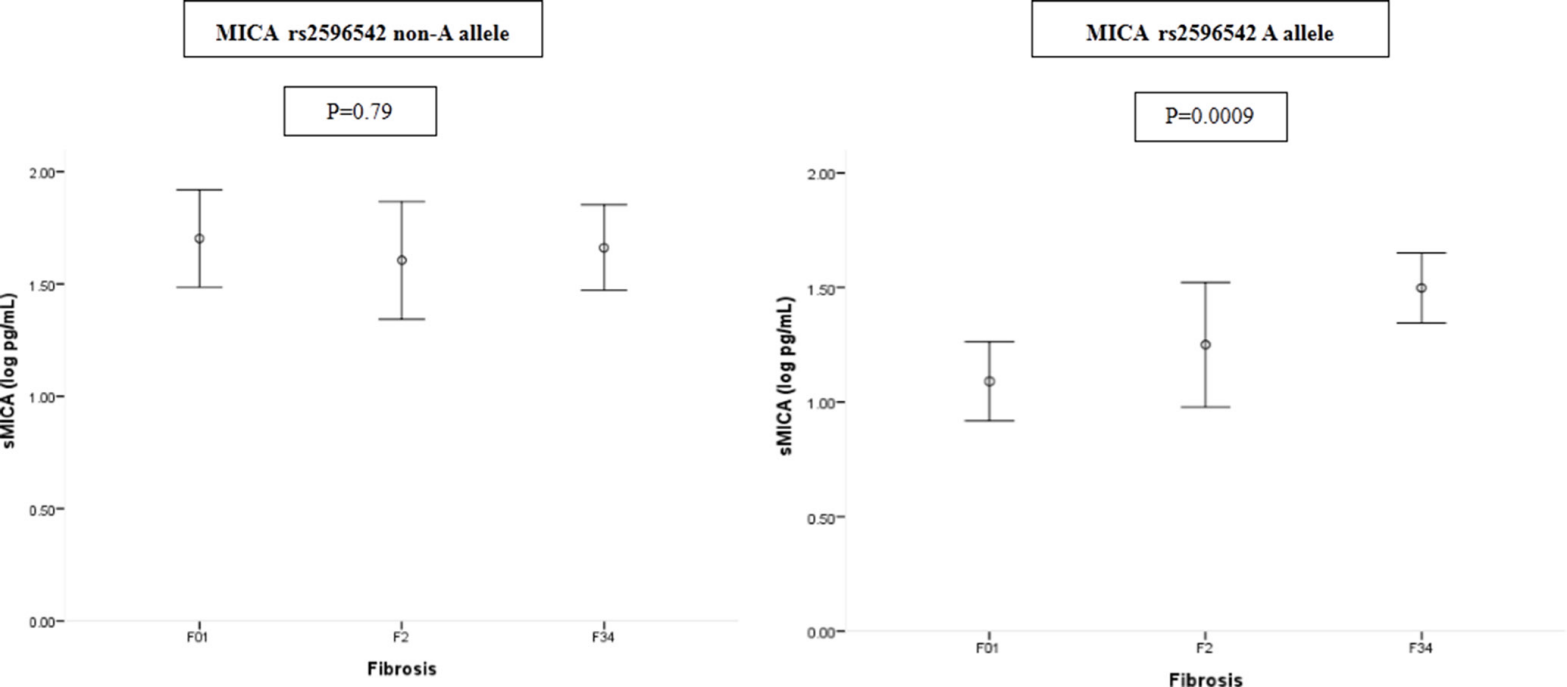
Serum MICA levels in patients with different fibrotic stages stratified by MICA rs2596542 single nucleotide polymorphism MICA, MHC class I polypeptide-related chain A.

Linear regression analysis revealed that factors independently correlated to sMICA were α-fetoprotein (β: 0.149; 95% confidence interval [CI]:0.001, 0.003; *P* = 0.007) and the MICA rs2596542 GG genotype (β: 0.209; 95% CI: 0.153, 0.483; *P* < 0.001). The MICA SNP was the strongest factor correlated with sMICA levels, and we further explored the factors correlated with sMICA in patients with different MICA SNPs. α-fetoprotein was independently correlated to sMICA in MICA rs2096542 non-A allele carriers ((β: 0.158; 95 % CI: 0.000, 0.003; *P* = 0.049). On the other hand, advanced fibrosis (F34) was the only factor independently correlated to sMICA among MICA A allele carriers (β: 0.234; 95% CI: 0.107, 0.543; *P* = 0.004) (Table 2). There was a significantly increased trend of sMICA levels from mild to severe fibrotic stages among the A allele carriers (*P* = 0.0009) but not the non-A allele (*P* = 0.79) carriers (Figure 1).

**Table 2:** Linear regression analysis of factors associated with serum MICA level

	B	Standard error	95% confidence intervals for B		Beta	*P* value
**All patients**						
α-fetoprotein	0.002	0.001	0.001	0.003	0.149	0.007
MICA rs2596542 GG genotype	0.318	0.084	0.153	0.483	0.209	< 0.001
**MICA non-A allele carriers**						
α-fetoprotein	0.002	0.001	.000	0.003	0.158	0.049
**MICA A allele carriers**						
F34	0.325	0.11	0.107	0.543	0.234	0.004

Note: Variables including age, sex, body weight, HCV genotype (1 vs. non-1), HCV viral loads, diabetes (yes vs. no), fibrosis (F0-2 vs. F34), platelet, α-fetoprotein,aspartate aminotransferase, alanine aminotransferase, *r*-glutamyl transferase.MHC class Ipolypeptide-related chain Ars2596542 GG genotype(A vs. non-A allele carriage). Odds ratio are for MICArs2596542 GG genotype(A vs. non-A allele carriage), α-fetoprotein (per 1 ng/mL increase), and fibrosis (F34 vs. F0-2).

### Levels of sMICA in predicting liver fibrosis in MICA rs2596542 A allele carriers

Serum MICA levels correlated with liver disease severity only in MICA rs2596542 A allele carriers. We further evaluate different cut-off values of sMICA in predicting liver fibrosis in the subpopulation. As shown in Tble 3, detectable sMICA (> 5.18 pg/mL) or sMICA > 50 pg/mL could differentiate patients with different degrees of liver fibrosis. sMICA > 50 pg/mL provided a positive predictive value of 72% in predicting advanced liver fibrosis (F34) and of 90% in significant fibrosis (> F2). The different cut-off values of APRI and FIB4 in predicting liver fibrosis defined by WHO [9] were also compared in Table 3.

**Table 3:** Predictive values of sMICA in predicting liver fibrosis for MICArs2596542 A allele carriers

	Non-advanced fibrosis F0-2 (*N* = 73) *n* (%)	Advanced fibrosis F34 (*N* = 84) *n* (%)	*P* value	SEN	SPE	PPV	NPV
				%	%	%	%
sMICA detectable	27 (37.0)	53 (63.1)	< 0.001	63	63	66	60
sMICA > 50 pg/mL	16 (21.9)	41 (48.8)	< 0.001	49	78	72	57
sMICA > 100 pg/mL	11 (15.1)	23 (27.4)	0.06	27	85	68	50

	Non-significant fibrosis F01 (*N* = 45) *n* (%)	Significant fibrosis F2-4 (*N* = 112) *n* (%)	*P* value	SEN	SPE	PPV	NPV
				%	%	%	%
sMICA detectable	16 (35.6)	64 (57.1)	0.01	57	64	80	38
sMICA > 50 pg/mL	6 (13.3)	51 (45.5)	< 0.001	46	87	90	39
sMICA > 100 pg/mL	5 (11.1)	29 (25.9)	0.04	26	89	85	33
FIB4 > 3.25*	9 (20.0)	65 (58.0)	< 0.001	58	80	88	43
APRI > 1.5*	14 (31.1)	70 (62.5)	< 0.001	63	69	83	43

	Non cirrhosis F0-3 (*N* = 95) *n* (%)	Cirrhosis F4 (*N* = 62) *n* (%)	*P* value	SEN	SPE	PPV	NPV
				%	%	%	%
sMICA detectable	42 (44.2)	38 (61.3)	0.04	61	56	48	69
sMICA > 50 pg/mL	28 (29.5)	29 (46.8)	0.03	47	71	51	67
sMICA > 100 pg/mL	17 (17.9)	17 (27.4)	0.16	27	82	50	63
APRI > 2^*^	34 (35.8)	32 (51.6)	0.05	52	64	49	67

Note: MICA:MHC class Ipolypeptide-related chain A.Detectable sMICA: > 5.18 pg/mL. APRI: the aspartate aminotransferase-to-platelet ratio index.FIB-4:fibrosis index based on four factors.* Cut-off values of FIB4 and APRI defined by WHO.

### Factors associated with advanced liver disease in patients with or without MICA rs2596542 A allele carriage

A univariate analysis of factors associated with advanced liver fibrosis in the entire population included an older age, a lower platelet count, and higher aspartate aminotransferase, *r*-glutamyl transferase, and α-fetoprotein levels (Table 1). A logistic regression analysis of the factors that were associated with advanced liver fibrosis included platelet counts (OR/CI:0.987/0.982–0.991, *P* < 0.001) and r-GT (1.005/1.000–1.009, *P* = 0.04). The result was similar to that of the patients with MICA non-A allele carriage, of whom factors independently associated with advanced liver fibrosis were platelet counts (OR/CI:0.986/0.979–0.993, *P* < 0.001) and r-GT (1.011/1.003-1.019, *P* = 0.009) (Table 4 and Table 5). On the other hand, a univariate analysis of factors associated with advanced liver fibrosis among MICA A allele carriers included an older age, lower platelet counts, and higher AST, AFP and sMICA levels (Table 4). A logistic regression analysis of the factors associated with advanced liver fibrosis included sMICA (OR/CI:2.996/1.428–6.287, *P* = 0.004) and platelet counts (OR/CI:0.988/0.982–0.994, *P <* 0.001) (Table 5).

**Table 4:** Univariate analysis of factors associated with advanced liver fibrosis in patients with different MICA rs2596542 SNP

	MICA rs2596542 non-A allele carriers			MICA rs2596542 A allele carriers		
	F0-2 (*n* = 89)	F3-4 (*n* = 73)	*P* value	F0-2 (*n* = 73)	F3-4 (*n* = 84)	*P* value
Age (years, mean ± SD)	51.4 ± 11.9	55.2 ± 8.5	0.02	50.6 ± 12.0	54.3 ± 11.6	0.049
Male gender, *n* (%)	45 (50.6)	35 (47.9)	0.74	40 (54.8)	45 (53.6)	0.88
Body weight (kg, mean ± SD)	65.2 ± 12.4	68.2 ± 11.9	0.13	66.5 ± 10.3	67.7 ± 12.3	0.51
Diabetes, *n* (%)	13 (14.6)	16 (21.9)	0.11	10 (13.7)	17 (20.2)	0.28
Platelet count (× 10^3^ *u*/L, mean ± SD)	183 ± 59	136 ± 58	< 0.001	184 ± 65	137 ± 57	< 0.001
AST (IU/L, mean ± SD)	91 ± 51	107 ± 54	0.047	102 ± 57	116 ± 71	0.18
ALT (IU/L, mean ± SD)	141 ± 92	143 ± 68	0.92	163 ± 101	154 ± 117	0.62
*r*-GT (U/L,mean ± SD)	51 ± 36	86 ± 73	< 0.001	67 ± 66	74 ± 49	0.45
α-fetoprotein (ng/ml, mean ± SD)	9.9 ± 13.2	40.2 ± 115.2	0.02	12.8 ± 16.8	24.0 ± 36.1	0.02
HCV genotype 1, *n* (%)	63 (70.3)	43 (58.9)	0.11	48 (65.8)	56 (66.7)	0.9
HCV viral loads (log IU/mL, mean ± SD)	5.71 ± 0.97	5.56 ± 0.91	0.29	5.54 ± 1.00	5.38 ± 0.94	0.33
MICA rs2596542 A allele, *n* (%)	-	-	-	-	-	-
sMICA (log pg/mL. mean ± SD)	1.67 ± 0.78	1.67 ± 0.82	0.96	1.15 ± 0.63	1.50 ± 0.70	0.001
sMICA > 50 pg/mL, *n* (%)	50 (56.2)	40 (54.8)	0.86	16 (21.9)	41 (48.8)	<0.001

Note: HCV, hepatitis C virus;MICA: MHC class Ipolypeptide-related chain A;sMICA: serum MICA level; *r*-GT: *r*-glutamyl transferase; SD: standard deviation; DM: diabetes mellitus; AST: aspartate aminotransferase; ALT: alanine aminotransferase.

**Table 5:** Logistic regression analysis of factors associated with advanced liver disease

Variables	OR	95% C.I.	*P* value
**All patients**			
Platelet count			
Per 1 *u*/Lincrease	0.987	0.982–0.991	< 0.001
*r*-GT			
Per 1 U/L increase	1.005	1.000–1.009	0.04
**MICA rs2596542 non-A allele carriers**			
Platelet count			
Per 1 *u*/L increase	0.986	0.979–0.993	< 0.001
*r*-GT			
Per 1 U/L increase	1.011	1.003–1.019	0.009
**MICA rs2596542 A allele carriers**			
Platelet count			
Per 1 *u*/L increase	0.988	0.982–0.994	< 0.001
sMICA			
< 50 pg/mL	1		0.004
> 50 pg/mL	2.996	1.428–6.287	

Note: MICA: MHC class Ipolypeptide-related chain A.sMICA: serum MICA level; *r*-GT: *r*-glutamyl transferase.

## DISCUSSION

Until now, studies regarding the association of MICA SNP and its serum levels with liver disease mainly focus on HCC [6–8, 10]. The correlation with HCV related liver fibrosis has never been discussed. We demonstrated that sMICA level was correlated to HCV related liver disease severity. However, the association was only restricted to MICA rs2596542 A allele carriers. There was an increasing trend of sMICA levels with the progression of liver fibrosis among A allele carriers. Furthermore, higher sMICA was independently associated with advanced liver fibrosis, and nine-tenths of the A allele carriers possessed significant liver fibrosis if their sMICA level was > 50 pg/mL.

The carriage of MICA rs2596542 A allele had low MICA production [6, 8]. The low affinity of NKG2D on natural killer (NK) cells is postulated to be the cause of HCC in CHC [8]. However, escaping immune surveillance may take place if soluble MICA in the circulation is overproduced, which might down-regulate NKG2D expression and disturb NKG2D-mediated antitumor immunity. As a consequence, the high levels of sMICA have been associated with a variety of malignancies, including melanoma, breast, colon, and HCC [11, 12]. However, evidence for the association of MICA SNP rs2596542 and sMICA with liver fibrosis is sparse. Kumar et al. have observed a higher sMICA level in cirrhotic CHC patients compared to uninfected healthy controls. However, they did not identify any differences between CHC patients with or without liver cirrhosis [8]. In line with previous reports [6, 8], the carriage of the rs2596542 GG genotype was the strongest factor correlating with sMICA level. The association of sMICA with liver fibrosis should be discussed separately in patients who carry different MICA SNPs. In the current study, neither the MICA SNP nor sMICA were associated with advanced liver disease among the whole population. Nevertheless, sMICA levels were independently associated with fibrosis in A allele carriers. Unlike GG genotype carriers whose sMICA were secreted abundantly regardless liver disease severity, sMICA increased in proportion to the liver fibrotic stages in A allele carriers. The actual pathophysiological mechanism is unknown, whether the shedding protein of MICA contributes interactively to fibrogenesis (ex. A Disintegrin And Metalloprotease, ADAM [13]) awaits further exploration.

In the current study, we demonstrated higher sMICA levels were independently associated with advanced liver disease in A allele carriers. A allele carriers with sMICA > 50 pg/mL had a three-fold risk of advanced liver fibrosis, including liver cirrhosis (F34), compared to their counterparts. The recommended treatment guidelines have proposed patients with F2 should be justified as a treatment priority [14, 15]. We concordantly proved a higher HCC risk in Taiwanese CHC patients with fibrotic stage 2 if they were left untreated [6]. WHO has advocated that CHC patients whose fibrotic stage is equal or greater than two have “significant fibrosis” [9]. In the current study, we identified a 90 % PPV of significant fibrosis in A allele carriers whose sMICA > 50 pg/mL. APRI and FIB4 are now widely adapted as non-invasive markers for predicting liver fibrosis.^9^ The predictive power of sMICA was not inferior to and even substantially better than other two surrogates in terms of positive predictive value or specificity, which may provide insight for the individualized application on the basis of host genetics.

The current study was limited due to its cross sectional design. We failed to demonstrate the influence of the genetics in liver disease progression by a longitudinal observation. The application of biomarker is genetic-dependent and may not be generalized to all patients. However, we have identified the clinical utility for predicting liver disease by measuring sMICA. The genetic test of MICA may facilitate the application of surrogate markers for HCC as well as liver fibrosis. In conclusion, serum MICA levels were highly correlated to liver disease severity in CHC patients who carried the MICA rs738409 A allele. The current study might shed light for further fibrogenesis molecular experiments as well as potential antifibrotic agents development. Studies upon other ethnicities or etiologies of liver disease are warranted to validate the current finding.

## MATERIALS AND METHODS

Eligible patients were biopsy-proven CHC patients who planned to undergo antiviral therapy in a medical center in Taiwan. Patients were excluded if they were co-infected with HIV or hepatitis B virus (HBV) infection. Patients were also excluded if they had the following diseases: autoimmune hepatitis, primary biliary cirrhosis, a current or past history of alcohol abuse (≥ 20 g daily), previous liver transplantation, or a past history of HCC. Anti–HCV antibodies were detected using a third-generation, commercially available enzyme-linked immunosorbent assay kit (AxSYM 3.0, Abbott Laboratories, Chicago, IL, USA). The biochemical items were measured on a multichannel auto analyzer (Hitachi Inc. Tokyo, Japan). Serum HCV RNA was detected using qualitative real-time polymerase chain reaction (PCR) (COBAS AMPLICOR Hepatitis C Virus Test, ver. 2.0; Roche, Branchburg, NJ, USA, detection limit: 50 IU/ml) or quantification branched DNA assay (Versant HCV RNA 3.0, Bayer, Tarrytown, New Jersey, USA; quantification limit: 615 IU/ml) if evaluated before 2011. The HCV genotypes were determined using the Okamoto method before 2011 [17]. After 2011, both the HCV RNA and genotype were detected using real-time PCR assays (Real Time HCV; Abbott Molecular, Des Plaines, IL, USA; detection limit: 12 IU/ml) [18]. The liver histology was graded and staged according to the scoring system described by Scheuer [19]. Liver disease severity, including significant fibrosis (> F2)^9^, advanced liver fibrosis (F34)^2^, and cirrhosis (F4), was judged for its association with sMICA. The study was approved by the ethics committees at the participating hospitals and was performed according to the guidelines of the International Conference on Harmonization for Good Clinical Practice. All patients gave written informed consent before enrollment.

### MICA rs2596542 genotyping and sMICA measurement

SNP rs2596542 of MICA was determined by ABI TaqMan^®^ SNP genotyping assays (Applied Biosystems, Foster City, CA, USA) using pre-designed, commercial genotyping assays (ABI Assay ID: C__27301153_10). Briefly, PCR primers and two allelic-specific probes were designed to detect a specific SNP target. PCR reactions were performed in 96-well microplates with ABI 7500 real-time PCR. Allele discrimination was achieved by detecting fluorescence using System SDS software version 1.2.3. The allele and genotype frequencies were consistent with Hardy-Weinberg equilibrium. sMICA was measured by a sandwich enzyme-linked immunosorbent assay (DuoSet MICA eELISA kits, R & D Systems, Minneapolis, MN, USA).

### Statistical analyses

The frequency was compared between groups using the χ^2^ test with the Yates correction or using Fisher's exact test. Group means, presented as the mean values and standard deviations, were compared using analysis of variance and Student's *t* test or the Mann-Whitney *U* test. The serum HCV RNA levels were expressed after logarithmic transformation of the original values. The aspartate aminotransferase (AST)-to-platelet ratio index (APRI) was calculated by the following equation: (AST level/upper limit of normal range)/platelet counts (10^9^/L) × 100. Fibrosis index based on four factors (FIB-4) was calculated as age ([yr] × AST [U/L])/((PLT [10(9)/L]) × (ALT [U/L])(1/2)). MICA SNP rs2596542 A allele carriage has been a risk factor of HCC in CHC [8]. The influence of the MICA SNP on liver fibrosis was calculated using a dominant (genotype GG vs. AG/AA) genetic model of inheritance. A stepwise logistic regression analysis was performed to evaluate the independent factors associated with advanced liver fibrosis by analyzing co-variants with *P* values < 0.05 in the univariate analysis. The statistical analyses were performed using the SPSS 12.0 statistical package (SPSS, Chicago, IL, USA). All statistical analyses were based on two-tailed hypothesis tests with a significance level of *P* < 0.05.

## Abbreviations

ALT: alanine aminotransferase
AST: aspartate aminotransferase
APRI: the aspartate aminotransferase-to-platelet ratio index
AFP: α-fetoprotein
CHC: chronic hepatitis C
GWAS: genome-wide association study
HCC: hepatocellular carcinoma
HCV: hepatitis C virus
MICA: MHC class I chain-related A
SNP: single-nucleotide polymorphism
